# Can near real-time monitoring of emergency department diagnoses facilitate early response to sporadic meningococcal infection? - prospective and retrospective evaluations

**DOI:** 10.1186/1471-2334-10-309

**Published:** 2010-10-27

**Authors:** Libby O'Toole, David J Muscatello, Wei Zheng, Tim Churches

**Affiliations:** 1 Australian Bureau of Statistics, Tasmanian Regional Office, 200 Collins Street, Hobart TAS 7000, Australia; 2 Centre for Epidemiology and Research, New South Wales Department of Health, 73 Miller Street, North Sydney NSW 2059, Australia

## Abstract

**Background:**

Meningococcal infection causes severe, rapidly progressing illness and reporting of cases is mandatory in New South Wales (NSW), Australia. The NSW Department of Health operates near real-time Emergency Department (ED) surveillance that includes capture and statistical analysis of clinical preliminary diagnoses. The system can provide alerts in response to specific diagnoses entered in the ED computer system. This study assessed whether once daily reporting of clinical diagnoses of meningococcal infection using the ED surveillance system provides an opportunity for timelier public health response for this disease.

**Methods:**

The study involved a prospective and retrospective component. First, reporting of ED diagnoses of meningococcal infection from the ED surveillance system prospectively operated in parallel with conventional surveillance which requires direct telephone reporting of this scheduled medical condition to local public health authorities by hospitals and laboratories when a meningococcal infection diagnosis is made. Follow-up of the ED diagnoses determined whether meningococcal infection was confirmed, and the time difference between ED surveillance report and notification by conventional means. Second, cases of meningococcal infection reported by conventional surveillance during 2004 were retrospectively matched to ED visits to determine the sensitivity and positive predictive value (PPV) of ED surveillance.

**Results:**

During the prospective evaluation, 31 patients were diagnosed with meningococcal infection in participating EDs. Of these, 12 had confirmed meningococcal disease, resulting in a PPV of 38.7%. All confirmed cases were notified earlier to public health authorities by conventional reporting.

Of 149 cases of notified meningococcal disease identified retrospectively, 130 were linked to an ED visit. The sensitivity and PPV of the ED diagnosis for meningococcal infection was 36.2% and 36.7%, respectively.

**Conclusions:**

Based on prospective evaluation, it is reassuring that existing mechanisms for reporting meningococcal infection perform well and are timely. The retrospective evaluation found low sensitivity and PPV of ED diagnoses for meningococcal disease. Even if more rapid forwarding of ED meningococcal diagnoses to public health authorities were possible, the low sensitivity and PPV do not justify this. In this study, use of an ED surveillance system to augment conventional surveillance of this scheduled medical condition did not demonstrate a benefit.

## Background

Infection by *Neisseria meningitidis *(meningococcus), causes rapidly progressing and potentially fatal systemic disease [1] and is a serious public health problem in many parts of the world. On average, five to ten percent of meningococcal infections are fatal and even among survivors, the other outcomes can also be devastating, with ten to 20 per cent developing permanent brain damage and disability [2]. Therefore, early identification of meningococcal cases can be life saving and is crucial for limiting further spread of the disease. Real-time surveillance of emergency department (ED) (or "emergency room") presentations may be able to complement traditional communicable disease surveillance systems by providing information on either sporadic cases or clusters of meningococcal disease presenting to EDs. To our knowledge, there have not been any reported studies on the sensitivity and positive predictive value of diagnoses recorded in ED patient management databases for meningococcal infection.

Meningococcal infection has been continuously notifiable since 1979 in Australia. In 1994, the Australian Meningococcal Surveillance Programme was established to monitor and analyse isolates of *Neisseria meningitidis *from cases of invasive meningococcal disease in Australia. Despite a steady decrease in the incidence of meningococcal infection since surveillance began, there were still 405 cases reported in 2004, representing a rate of 2 per 100,000. In the state of New South Wales (NSW), Australia, approximately 150 to 200 new meningococcal cases are reported each year, most commonly in young children, adolescents and young adults. The overall NSW rate in 2007 was 1.6 per 100,000 [3]. Meningococcal disease is a scheduled medical condition and notification of meningococcal infections to regional public health authorities is a legal requirement in NSW.

In 2003, Centre for Epidemiology and Research of the NSW Department of Health, which is the state government health authority, implemented a syndromic surveillance system that uses near real-time, de-identified data from patient management information systems used in public hospital emergency departments [5]. Rapid availability of ED visit information may offer timelier identification and reporting of ED visits assigned a diagnosis of meningococcal infection.

We conducted a study to evaluate the sensitivity and positive predictive value (PPV) of meningococcal diagnoses from EDs and whether daily reporting of new meningococcal visits offered a time advantage.

## Methods

The evaluation comprised two components, a prospective and a retrospective evaluation. The prospective evaluation used data from the ED surveillance system. It aimed to determine the sensitivity and PPV of ED diagnoses of meningococcal infection for detecting true positive meningococcal cases and whether the reporting based on the ED diagnosis of meningococcal infection was timelier than conventional notifications. The retrospective evaluation used data collected in the NSW ED and notifiable disease databases. The aim was to provide a more representative estimate of the sensitivity and PPV of ED diagnoses of meningococcal infection. Specificity and negative predictive value were not estimated because among the population of all ED visits, true negatives would be very large in number, giving a specificity and NPV close to 100%.

### Prospective evaluation

#### Data source

In 2005, there were 15 EDs in the Sydney metropolitan area participating in the near real-time ED surveillance system, representing 52% of ED visits in the greater Sydney metropolitan area. Selected information recorded in patient management databases at the EDs is transmitted either in real-time or in four or six-hourly batch files to a surveillance database at the NSW Department of Health. Variables include age, sex, postal code of residence, presenting problem, nursing assessment text from triage, triage acuity, one or more provisional ED diagnoses, and discharge status [5]. The routine ED surveillance reporting system automatically prepares statistical reports that highlight unusual trends in a range of acute health problems. On detection of unusual disease activity, situation reports are sent by surveillance personnel using electronic email to relevant Departmental and regional public health authorities for consideration with other surveillance intelligence.

#### Study design and data collection

Commencing 1 November 2004, apart from the routine ED surveillance reporting, an additional reporting system was established for evaluating the reporting of ED diagnoses of meningococcal infection. Once daily, information on any ED visits assigned a first or additional ED diagnosis of meningococcal infection that had been received in the surveillance database in the previous 24 hours was extracted and ED surveillance personnel at the NSW Department of Health reported the information by telephone to the relevant regional public health authority. The visits were identified from the 15 EDs participating in the syndromic surveillance system at that time. Meningococcal diagnoses were identified using International Classification of Diseases (ICD) version 9 code 036 and ICD version 10 code A39. The regional public health authority was chosen based on the patients' postal code of residence.

State ED surveillance personnel were alerted to the occurrence of meningococcal diagnoses in the reports by an automatically generated email. These emails were available for checking seven days per week as one of the first daily duties for syndromic surveillance personnel.

The regional public health authority was contacted by ED surveillance personnel one week after each initial telephone report to determine whether meningococcal infection was confirmed and to conduct a brief follow-up telephone interview. The interview determine whether the patient had been notified by conventional scheduled medical condition reporting, when that notification occurred, and whether the ED surveillance report offered additional information beyond that provided by the conventional notifier.

After 9 months, the interim analysis reported here identified that there was no benefit in continuing the prospective evaluation and, following consultation with stakeholders, reporting ceased on 22 July 2005.

### Retrospective Evaluation

#### Data sources

The NSW ED Data Collection is a state database [6] managed by the Demand and Performance Evaluation Branch of the NSW Department of Health and derived from the same ED public hospital patient management information systems from which the ED syndromic surveillance system obtains its information. The difference is that the state-wide data collection is updated weekly or monthly and cannot therefore be used for near real-time surveillance. In 2004, there were 57 EDs participating in the data collection, including most urban and larger rural public hospitals in NSW. These hospitals received approximately three quarters of all NSW public hospital ED visits. Almost all ED services in NSW are provided by public hospitals. The data collection records a broader range of data items compared to the syndromic surveillance system, and includes identifying information, such as the patient's name and full residential address.

The NSW notifiable diseases database is a state data collection managed by the Communicable Disease Branch of the NSW Department of Health. It records all scheduled communicable disease infections in NSW, including meningococcal infection. In 1991, it became mandatory that hospitals and laboratories notify their local public health authority as soon as possible after a provisional diagnosis of meningococcal disease was made [4]. The aim of reporting to public health authorities is the prevention of further spread of the disease through follow-up of case contacts and prophylactic administration of antibiotics where necessary. Both probable meningococcal cases, based on clinical evidence, and confirmed meningococcal cases, based on positive laboratory test results, are recorded in the database by regional public health authority staff as soon as the case is notified. The probable cases are removed within one working day after an alternative diagnosis is made. Information captured in the notifiable diseases database includes demographic information, disease notified, name of the pathogen as well as identifying information including, for some infections, the patient's name and full residential address.

#### Study design and data collection

For calendar year 2004, all confirmed meningococcal cases were extracted from the notifiable diseases database and the resulting records were then matched against ED visits reported in the ED data collection by using a sequential deterministic data linkage method [7]. This year was chosen because it was the most recent full calendar year for which data were available in both databases. The identifiers used for matching included the patient's full name, date of birth, age, sex, postcode of residence and medical record number. A meningococcal notification in the notifiable diseases database was considered the gold standard. Matched records were classified as true positives if the ED visit record contained any diagnosis of meningococcal disease. ED visits with other diagnoses were treated as false negatives. ED visits with a diagnosis of meningococcal disease in the ED data collection that were not located in the notifiable diseases database were classified as false positives. If all completed variables matched but some variables were missing, the ED and notifiable disease records were treated as a match.

Matching was done automatically using SAS Software version 8 [8] and the results were manually reviewed to ensure appropriate matching and exclusions. Since patients may present to an ED more than once for the same condition, only the earliest ED visit date on or after the notified disease onset date was used. To prevent classification and observer bias, matching of ED visits was completed with the investigator (LO) blinded to the ED diagnosis and visit details. These details were revealed only when matching was complete by joining the matched components back to the original records.

### Statistical analysis

Sensitivity was estimated as the ratio of true positive ED visits to the sum of true positive and false negative ED visits. PPV estimates were calculated as the ratio of true positive ED visits to all positive ED visits (true positives + false positives). We calculated 95% confidence intervals (CI) as for a proportion, using the normal approximation for a binomial variable: $p+/-1.96\sqrt{p(1-p)/n}$[9,10].

As employees of the NSW Department of Health involved in the operation and management of public health surveillance systems, the authors had authorised access to both data sources which are collected, managed and used by the organisation in accordance with the NSW Health Administration Act 1982 [11], the NSW Public Health Act 1991 [12], and the NSW Health Records and Information Privacy Act 2002. This quality assurance study was conducted under the health services management provisions of the Health Records and Information Privacy Act 2002 (New South Wales) [13].

## Results

### Prospective Evaluation

During the prospective evaluation from 1 November 2004 until 22 July 2005, 31 patients presenting to any of the 15 NSW EDs were subsequently assigned a provisional diagnosis of meningococcal disease and were reported to local public health authorities. Of these, 12 were confirmed to have meningococcal infection, providing a PPV of 38.7% (95% CI: 21.6% to 55.9%).

Patients diagnosed with meningococcal disease in the ED who did not go on to be a confirmed case were, in the large part, presenting with symptoms in the meningococcal disease spectrum. All 12 true positive cases were notified to public health authorities prior to the ED surveillance staff notification, mostly by the treating clinicians from the hospital (9, 75%). ED surveillance data did not provide additional information to that routinely notified to the regional public health authorities under conventional scheduled medical condition reporting.

### Retrospective Evaluation

In 2004, the notifiable diseases database recorded 149 notifications of meningococcal disease. Children aged 0 to 4 years constituted about one-third (51, 34%) of the notified cases, while persons aged 15 to 24 years constituted one-fifth (30, 20%) (Table 1). In the same period, 147 patients were assigned a provisional ED diagnosis of meningococcal disease in any of the EDs included in the ED data collection. Children aged 0-4 years constituted nearly half of those visits (65, 44%) while 15-24 year-olds constituted only 9%. The second largest group was 5-14 year olds (35, 24%). For both ED visits and notifiable diseases database notifications, males were slightly overrepresented (Table 1).

**Table 1:** Demographic data for notifications of confirmed meningococcal cases and emergency department diagnoses of meningococcal disease, NSW, 2004

	Confirmed meningococcal cases		ED diagnoses of meningococcal disease	
Age groups	N	%	N	%
*0 to 4 years*	51	34	65	44
*5 to 14 years*	17	11	35	24
*15 to 24 years*	30	20	13	9
*25 to 44 years*	18	12	21	14
*44+ years*	33	22	13	9
				
**Sex**				
*Male*	76	51	80	54
*Female*	73	49	67	46
				
**Total**	**149**	**100**	**147**	**100**

Of the meningococcal cases recorded in the notifiable diseases database, 130 (87%) were found to have a match in the ED data collection. Of 130 matched cases, 54 were assigned a provisional diagnosis of meningococcal disease ("true positives") and the remaining 76 visits were diagnosed with a different, or less specific condition including septicaemia, meningitis, fever and respiratory conditions ("false negatives"). There were 19 cases that failed to match to any ED visit, and were included in the false negative group. In addition, 93 ED visits were assigned a diagnosis of meningococcal disease but were not matched to a meningococcal case in the notifiable diseases database ("false positives") (Table 2).

**Table 2:** Emergency Department diagnoses of meningococcal disease and notifications of confirmed meningococcal cases, NSW, 2004 (retrospective evaluation)

	Confirmed meningococcal cases	Not notified as meningococcal case	Total
ED diagnosis of meningococcal disease	54	93	147
ED diagnosis other than meningococcal disease	95 (including 19 unmatched cases)	Unknown	N/A
**Total**	**149**	**N/A**	**N/A**

Sensitivity = 54/(54+95) = 36.2% (95%CI: 28.9%-44.5%)

Positive Predictive Values = 54/(54+93) = 36.7% (95%CI: 28.5%-44.0%)

The sensitivity of the ED visit provisional diagnosis was 36.2% for diagnoses of meningococcal disease (95% CI: 28.9% to 44.5%). The PPV of an ED provisional diagnosis of meningococcal disease was 36.7% (95% CI: 28.5% to 44.0%) (Table 2).

## Discussion

The prospective evaluation reassuringly demonstrated that conventional mechanisms for reporting meningococcal diseases from hospitals to public health authorities in NSW work well, with all true meningococcal cases being reported by hospitals or laboratories to local health authorities earlier than could be achieved with once daily checking for new meningococcal infection diagnoses in the ED surveillance database. Both the prospective and retrospective evaluations found a similar, low PPV of the ED meningococcal diagnoses of 37%. Sensitivity from the retrospective evaluation was also low, at 36%. Therefore, even if the ED surveillance system and procedures were changed to facilitate more rapid forwarding of meningococcal diagnoses to public health authorities, the low PPV and sensitivity combined with the high cost of public health resources devoted to each notification of this serious disease would not justify timelier ED reporting.

Several studies have explored the usefulness of ED data streams for complementing more traditional surveillance of various health problems [14-21]. They demonstrated that ED surveillance had the potential to identify increases in disease incidence at the population level earlier than traditional surveillance data sources. However, none of them specifically evaluated both the accuracy and timeliness of ED diagnoses for recognising a specific disease in individuals. There are, however, studies that evaluated the accuracy of hospital diagnoses for meningococcal infection or meningitis and encephalitis syndromes. Ackman *et al*. [22] evaluated hospital discharge diagnoses for patients admitted with meningococcal disease. Hospital coding of the diagnosis was completed using medical record abstraction by the hospital medical records department. One-third of the hospital meningococcal diagnoses were false positives. Of these, 70% were coding errors. However, the PPV of the primary hospital discharge diagnosis was 78%, much higher than in our study. The high PPV probably reflects the benefit of coding by personnel trained in medical coding and who have access to more complete medical records that include additional clinical results that may not be available during the ED episode of care. This differs from the ED diagnosis code in our setting that are selected by clinical or clerical ED personnel in the course of their work and who are generally not trained in medical coding. Gundlapalli *et al*. evaluated a computer-based surveillance system for early detection of meningitis and encephalitis syndromes using a variety of information available in an integrated hospital information system. In that study, ED diagnoses had a low positive predictive value of 39% for the syndromes [23]. This is consistent with our findings, although their study was not limited to syndromes caused solely by meningococcal infection.

Our study used a sound and comprehensive approach to evaluate several dimensions of surveillance of meningococcal disease using routinely collected ED information system data. Timeliness, reporting and response burden as well as the sensitivity and PPV of the surveillance system were considered. Since public health surveillance is a real-time pursuit, many aspects are difficult to reproduce retrospectively. Prospective study accurately records real clinical situations yet a retrospective evaluation allows greater analytical flexibility. This study has the advantage of both approaches.

In interpreting these results, there are a number of limitations to be considered. Firstly, linked data typically has a high false positive matching rate [7]. Due to the lack of a shared unique identifier in the linked data sets, we were unable to validate the linkage. Secondly, the "gold standard" we used is unlikely to include 100% of meningococcal cases. The NSW notifiable diseases database has its own, unknown sensitivity and specificity, and is thus not a true population-based gold standard.

The prospective evaluation only included a selected group of metropolitan EDs. Therefore, we cannot generalise the timeliness results to the remainder of NSW. Nevertheless, the similar PPV found in the retrospective evaluation indicates that the diagnostic tendency for meningococcal disease in the prospective evaluation was typical of the larger EDs across NSW that participate in the state ED Data Collection.

The low sensitivity indicates a tendency for ED clinicians to err on the side of caution when considering a provisional diagnosis of meningococcal disease. This is unsurprising, given the heightened public and media concern surrounding the disease and the potential legal implications of missed cases. The provisional diagnosis may also assist in ensuring that the necessary clinical services for the patient are made available.

The results of this study should not be interpreted as a limitation of ED syndromic surveillance generally. The NSW ED surveillance system was originally established for syndromic surveillance and uses groupings of ED diagnoses to categorise ED visits into syndromes. The syndrome time-series are automatically monitored using statistical methods to provide early warning of increasing incidence. Using the syndromes to identify single cases presenting with disease caused by a specific pathogen cannot be justified at this stage. Often firm diagnoses are not determined until after a patient with a serious illness has been discharged from the ED and admitted to a hospital ward.

## Conclusions

This study does not provide support for extending the scope of an ED-based syndromic surveillance system to include identification of scheduled medical conditions. For meningococcal disease, conventional notification procedures provide greater completeness and timeliness than daily extraction of ED diagnoses. Traditional notification systems should continue to be used for early public health alert of cases of meningococcal disease.

## Competing interests

The authors declare that they have no competing interests.

## Authors' contributions

LOT participated in the design of the study, carried out the data linkage, statistical analysis and drafted the manuscript. DM conceived of the study, and participated in its design and coordination and helped draft and finalise the manuscript. WZ assisted with statistical analysis and helped to draft and finalise the manuscript. TC advised on the design of the study and on data linkage, and edited drafts of the manuscript. All authors read and approved the final manuscript.

## Pre-publication history

The pre-publication history for this paper can be accessed here:

http://www.biomedcentral.com/1471-2334/10/309/prepub
